# RNA m^6^A Alterations Induced by Biomineralization Nanoparticles: A Proof-of-Concept Study of Epitranscriptomics for Nanotoxicity Evaluation

**DOI:** 10.1186/s11671-022-03663-x

**Published:** 2022-02-05

**Authors:** Jinbin Pan, Jiaojiao Wang, Kun Fang, Wenjing Hou, Bing Li, Jie Zhao, Xinlong Ma

**Affiliations:** 1 Department of Radiology, Tianjin Key Laboratory of Functional Imaging, Tianjin Medical University General Hospital, Tianjin, 300052 China; 2 Department of Orthopedics, Tianjin Hospital, Tianjin University, Tianjin, 300211 China; 3 Department of Radiology, The Second Hospital of Tianjin Medical University, Tianjin, 300211 China; 4 Department of Radiology, Beijing Chest Hospital, Capital Medical University, Beijing Tuberculosis and Thoracic Tumor Research Institute, Beijing, 101149 China; 5 Department of Diagnostic and Therapeutic Ultrasonography, Tianjin Medical University Cancer Institute and Hospital, National Clinical Research Center of Cancer, Key Laboratory of Cancer Prevention and Therapy, Tianjin’s Clinical Research Center for Cancer, Tianjin, 300060 China

**Keywords:** Epitranscriptomics, Nanotoxicity, RNA N^6^-methyladenosine, Nanomaterials, Biomineralization

## Abstract

Although various strategies have been included in nanotoxicity evaluation, epitranscriptomics has rarely been integrated into this field. In this proof-of-concept study, N6-methyladenosine (m^6^A) changes of mRNA in HEK293T cells induced by three bovine serum albumin (BSA)-templated Au, CuS and Gd_2_O_3_ nanoparticles are systematically explored, and their possible biological mechanisms are preliminarily investigated. It has been found that all the three BSA-templated nanoparticles can reduce m^6^A levels, and the genes with reduced m^6^A are enriched for TGF-beta signaling, which is critical for cell proliferation, differentiation and apoptosis. Further results indicate that abnormal aggregation of m^6^A-related enzymes at least partly account for the nanoparticle-induced epitranscriptomic changes. These findings demonstrate that epitranscriptomics analysis can provide an unprecedented landscape of the biological effect induced by nanomaterials, which should be involved in the nanotoxicity evaluation to promote the potential clinical translation of nanomaterials.

**Supplementary Information:**

The online version contains supplementary material available at 10.1186/s11671-022-03663-x.

## Introduction

Nanosafety is attracting considerable attention with the booming development and extensive applications of nanotechnology in the field of biomedicine [1, 2]. Although versatile nanomaterials with unique physiochemical characteristics are promising for diagnosis and therapy of diverse diseases, few nanoagents have been approved for clinical use due to the indistinct biosafety [3]. As a result, a standard and comprehensive nanotoxicological evaluation framework is highly desired for clarifying biosafety of nanoagents to guide their controlled synthesis and promote their clinical translation [4–6].

**Fig. 1:**
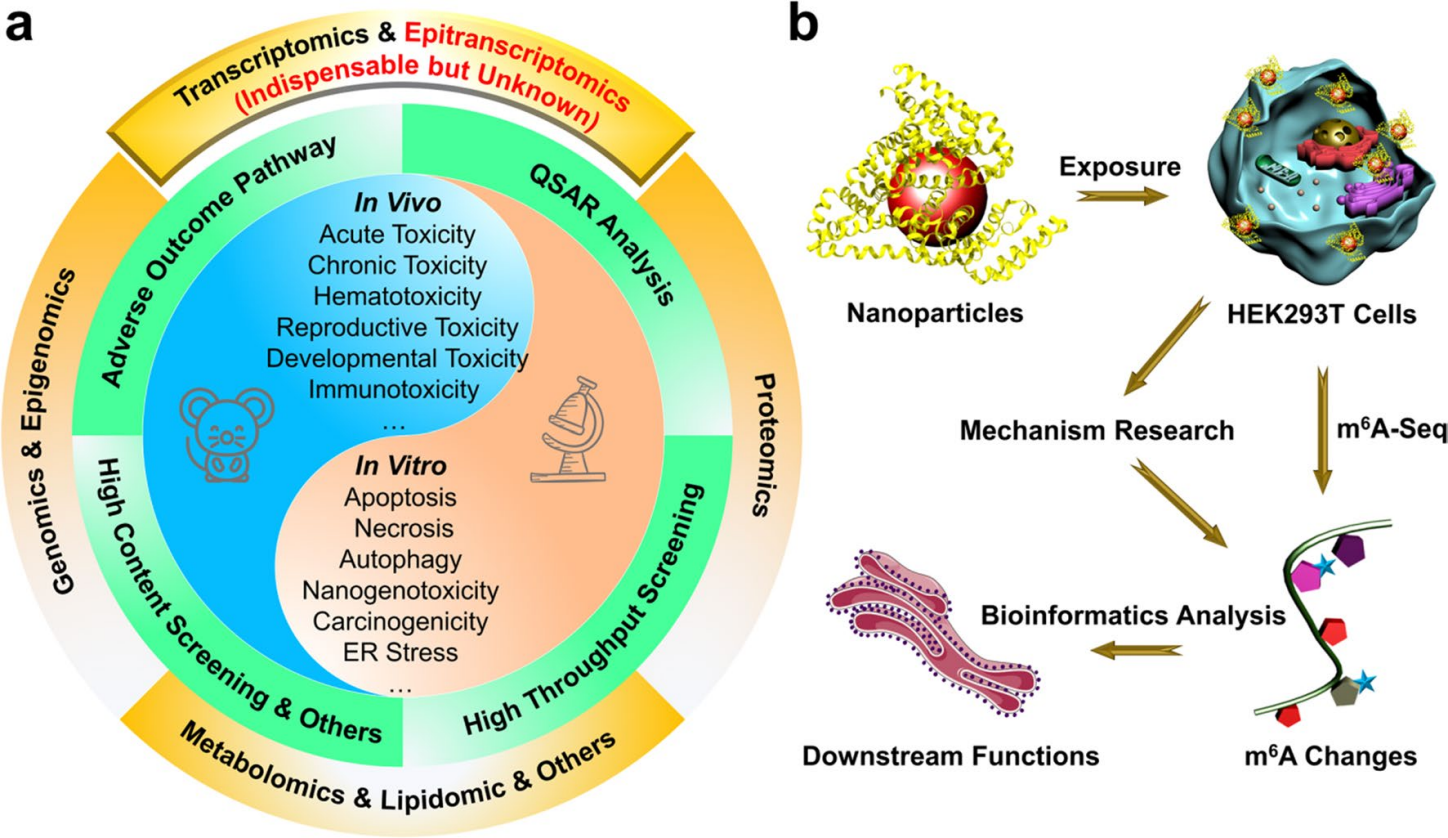
Global view of nanotoxicology. **a** Illustration of current nanotoxicity evaluation frameworks (QSAR: quantitative structure–activity relationship). **b** Schematic diagram of the epitranscriptomic nanotoxicity evaluation of BSA-templated NPs

In the past decade, various strategies have been developed to evaluate nanotoxicity from in vitro to in vivo (Fig. 1a) [7–18], and the adverse outcome pathway (AOP) framework and the quantitative structure–activity relationship (QSAR) modeling have been commonly used at the cellular level due to their simplified process, high efficiency, good logicality and predictability [8, 19–21]. However, these methods generally focus on specific genes, proteins, organelles and biological processes, which cannot provide a full picture of the nano-biological interactions [7]. In contrast, the emerging omics analysis can provide full characterization and quantification of biological effects of nanomaterials at a given molecular level, such as DNA, RNA, proteins, and lipids [22]. By integrating with bioinformatic analysis, the omics-based approaches are capable of mapping the toxicity-related molecular pathways and biological processes [23]. To date, nanotoxicology has entered into a new era following the development of various omics-based methods, such as genomics [24], epigenomics [25], transcriptomics [26, 27], proteomics [28], lipidomics [29, 30], and metabolomics [22, 31, 32].

Epitranscriptomics describes post-transcriptional RNA modifications that can dynamically regulate gene expression and control cell fate [33]. N^6^-methyladenosine (m^6^A) is the most abundant internal mRNA modification in eukaryotic cells, which functionally modulates the eukaryotic transcriptome to influence mRNA splicing, export, localization, translation and stability [34–37], as well as to regulate the expression of genes controlling extensive biological processes, such as development, reproduction, metabolism, immunity, and tumorigenesis [34, 38, 39]. Aberrant m^6^A as crucial drivers of multiple diseases (especially cancers) can provide an epitranscriptomic indicator of cellular responses and pathogenetic effects induced by nanomaterials, of which field remains largely unknown [40–43]. Although several omics-based approaches have been used to assess biological effects of nanomaterials, the epitranscriptomics has rarely been applied in the field of nanosafety evaluations so far [44]. Therefore, it is of great significance to investigate the nanomaterial-induced epitranscriptomics scenery.

Protein-templated biomineralization nanomaterials have attracted considerable interests in the field of biomedicine, and shown great potential in clinical translations due to their facile synthesis process, ultra-small and uniform size, remarkable colloidal stability and water solubility, good biocompatibility and favorable theranostics capability [45, 46]. In this proof-of-concept study, three types of bovine serum albumin (BSA)-templated nanoparticles (NPs) for different biological applications (BSA-Au NPs for fluorescent imaging, BSA-CuS NPs for photothermal therapy, and BSA-Gd_2_O_3_ NPs for magnetic resonance imaging) were employed to study the nanotoxicity in terms of epitranscriptomics. These NPs were synthesized by the biomineralization method, and m^6^A changes induced by these NPs were measured by the immunoprecipitation sequencing (m^6^A-seq). Functional annotation of genes with m^6^A changes were performed using the bioinformatic analysis, and biological mechanisms accounting for m^6^A changes were investigated via cell biological techniques (Fig. 1b). These results indicated that BSA-templated NPs could interfere the processing of m^6^A-related enzymes to reduce m^6^A level of diverse genes, which are relevant to multiple cellular pathways and biological processes. Therefore, epitranscriptomic effects (e.g., the m^6^A changes) induced by NPs were nonnegligible biological events, which should be integrated into the biosafety evaluation of nanomaterials for their potential clinical translation.

## Experimental

### Synthesis of BSA-Templated NPs

For the synthesis of BSA-Au NPs, 2 mL of aqueous HAuCl_4_ solution (25 mM, 37 °C) was added to 8 mL of BSA solution (250 mg, 37 °C) under vigorous stirring. Five minutes later, 0.5 mL of NaOH solution (1 M) was added, and the reaction was kept under vigorous stirring at 37 °C for 12 h.

For the synthesis of BSA-CuS NPs, 1 mL of aqueous Cu(NO_3_)_2_ solution (200 mM) was added to 7.5 mL of BSA solution (250 mg) under vigorous stirring. Five minutes later, 0.5 mL of NaOH solution (1 M) was quickly added. Then, 2 mL of Na_2_S (200 mM) was added and the reaction was kept under vigorous stirring at 90 °C for 0.5 h.

For the synthesis of BSA-Gd_2_O_3_ NPs, 0.5 mL of aqueous Gd(NO_3_)_3_ solution (100 mM) was added to 9.5 mL of BSA solution (250 mg) under vigorous stirring. Five minutes later, 0.5 mL of NaOH solution (1 M) was added, and the reaction was kept under vigorous stirring at room temperature for 2 h.

All these obtained BSA-templated NPs were purified by dialysis (molecular weight cut off: 8–14 kDa), and then freeze-dried and stored in dark at 4 °C for further use.

### Characterization of NPs

The size and morphology of BSA-templated NPs were determined on a Philips Tecnai G^2^ F20 (Philips, Holland) field emission transmission electron microscopy (TEM). The Fourier transform infrared (FT-IR) spectra (650–4000 cm^−1^) of BSA, BSA-Au, BSA-CuS, and BSA-Gd_2_O_3_ NPs were measured on a Nicolet iS10 spectrometer (Nicolet, USA) with background of pure KBr. Fluorescent spectra were recorded on a F7000 spectrofluorometer (Hitachi, Japan) equipped with a plotter unit and a quartz cell (1 cm × 1 cm). The absorption spectra were obtained via a UV-3600 plus spectrophotometer (Hitachi, Japan). The hydrodynamic size and zeta potential were determined on a Malvern Zetasizer (Nano series ZS, UK). The metal elements in nanoparticles were identified through the inductively coupled plasma-atomic emission spectrometer (ICP-AES, Thermo Fisher, ICAP 7400, USA).

### Storage Stability of NPs

To investigate the storage stability of BSA-templated metallic nanoparticles, the three NPs in freeze-dried powder state and aqueous state (4 mg/mL) were stored at 4 °C for 2 weeks, and their common state, optical absorption property, fluorescence property, magnetic resonance imaging capability, and hydrodynamic size were systematically evaluated at different timepoints (0, 7, and 14 days).

### Protein Corona Analysis

We preliminarily evaluated the protein corona formation in vitro by mixing the BSA-templated NPs (5 mg/mL) with 10% fetal bovine serum (FBS). Then, the mixing solutions were put into an oscillator under 37 °C, and the hydrodynamic sizes of solutions were monitored at different timepoints (0, 0.5, 1, and 3 h) post-mixing.

### Plasmids

The pFLAG-CMV vectors were provided by Sigma. Human METTL3 was cloned into pFLAG-CMV vectors by Gibson clone with forward primer ACAAGCTTGCGG CCGCGAATTCAatgtcggacacgtggag and reverse primer GGTCACAGGGATGCCACCCG GGATCCtaaattcttaggtttagag.

### Cell Culture and Transfection

HEK293T cells were obtained from American Type Culture Collection (ATCC) and grown in DMEM medium (Invitrogen) containing 10% FBS and 1% penicillin–streptomycin. Plasmids were transfected into cells with Lipofectamine 3000 (Invitrogen) according to manufacturer’s instructions.

### Cytotoxicity of NPs

The cytotoxicity of BSA-templated NPs was measured with HEK293T cells and 3T3-L1 cells via the standard Methyl Thiazolyl Tetrazolium (MTT) assays. Cells were seeded in 96-well culture plates at density of 1 × 10^4^ cells/well in 2 mL of DMEM supplemented with 10% FBS and 1% penicillin–streptomycin at 37 °C under 5% CO_2_ and cultured for 24 h. Then, the stale medium in each well was replaced with 2 mL of fresh medium containing different concentrations (50, 100, and 200 μg/mL) of NPs (BSA-Au, BSA-CuS, and BSA-Gd_2_O_3_ NPs), respectively. After another 24-h incubation, the cells were washed with PBS and treated with fresh medium containing MTT (0.25 mg/mL). Four hours later, the supernatant in each well was replaced with 120 μL of DMSO. After a mild shake for 10 min, the absorbance of each well at 490 nm was measured on a microplate reader (Bio-tek, USA). Then, the cell viability under the exposure of BSA-templated NPs was calculated.

To evaluate the influences of NPs on the apoptosis of cells, HEK293T cells were incubated in six-well culture plates at a density of 1 × 10^5^ cells per well in 200 μL of DMEM supplemented with 10% FBS and 1% penicillin–streptomycin at 37 °C under 5% CO_2_ and cultured for 24 h. Then, the stale medium in each well was replaced with 200 μL of fresh medium containing different concentrations (50, 100, and 200 μg/mL) of NPs (BSA-Au, BSA-CuS, and BSA-Gd_2_O_3_ NPs), respectively. After incubation for another 24 h, the apoptosis of HEK293T cells was evaluated by using Annexin V-FITC/PI Apoptosis Detection Kit (KeyGEN, Shanghai, China).

### Nanoparticles Uptaken by Cells

The nanoparticles uptaken rates by HEK293T cells were identified through the ICP-AES. Briefly, HEK293T cells were seeded in a 10 cm-culture dish at a density of 1 × 10^6^ cells/dish in 8 mL of DMEM supplemented with 10% FBS and 1% penicillin–streptomycin at 37 °C under 5% CO_2_, and cultured for 24 h. Then, 8 mL of fresh culture medium containing 200 μg/mL nanoparticles (BSA-Au, BSA-Gd_2_O_3_, and BSA-CuS NPs) was used to replace the old culture medium in each dish. After another 24-h incubation, the cells were washed with PBS, digested from the dish bottom with trypsin, and dispersed in PBS. After centrifugation at 1000 rpm for 3 min, and the supernatant was discarded. This washing process was repeated 3 times. Finally, these cells were fully dissolved with aqua regia, and the metal elements were quantified by ICP-AES. Cells cultured with 8 mL of fresh medium without nanoparticles were processed in the same way as the control group.

### m^6^A-seq and Data Analysis

m^6^A-seq was performed following previously reported protocol [47]. Total RNA was extracted by homogenizing cells in TRIzol reagent. mRNA was further purified using GenElute™ mRNA Miniprep Kit (Sigma). RNA fragmentation and m^6^A-immunoprecipitation were performed with Magna MeRIP™ m^6^A Kit according to the instructions. The library preparation and sequencing were carried out on Illumina HiSeq 2000 according to the manufacturer’s instructions. Samples were sequenced by with single-end 50-bp read length. All reads were mapped to human genome version hg19 by tophat v2.0.13 with default settings. The m^6^A level changes for nanoparticles/control were calculated by using exomePeak. Gene expression level changes for input and treatment were analyzed using Cuffdiff. The sequence reads were visualized with Integrative Genomics Viewer [48].

#### Redox-Western Blotting

Cells were lysed using high KCl lysis buffer and sonicated [49]. Equal amounts of proteins were loaded and separated by SDS-PAGE, transferred to polyvinylidene fluoride membranes, and detected by immunoblotting with the Millipore Immobilon Western Chemiluminescent HRP Substrate. Antibodies used for western blotting were as follows unless otherwise specified: METTL3, METTL14, WTAP, and FTO were bought from Cell Signaling Technology (CST), ALKBH5, YTHDC1, YTHDF2, and YTHDF3 were obtained from (ProteinTech), and FLAG (M2, F3165) was provided by Sigma-Aldrich. β-actin and β-tubulin (Santa Cruz) were used as loading controls.

#### RNA Isolation and Quantitative RT-PCR

Total RNA was isolated from cultured cells using TRIzol reagent. First-strand complementary DNA (cDNA) was synthesized by reverse transcription of 1 μg RNA using HiScript Q RT SuperMix for qPCR (+ gDNA wiper) (Vazyme). QPCR was carried out using ChamQ Universal SYBR qPCR Master Mix (Vazyme) and mRNA expressions were normalized to reference genes GAPDH. The primers used in all qPCR assays are listed in Additional file 1: Table S1 [50, 51].

#### MeRIP-qPCR

The MeRIP-qPCR was conducted as previous reported [52]. Briefly, total RNA was isolated with Trizol reagent, and then mRNA was further purified using GenElute™ mRNA Miniprep Kit (Sigma). Two μg of the purified mRNA was fragmentized into 100–200 nt length with fragmentation buffer at 94 °C for 5 min. The mRNA fragments were purified with Rneasy Mini Kit (QiaGen) and then subjected to immunoprecipitation with m^6^A antibody. After extensive wash, the immunoprecipitated fragments were eluted by competition using free N^6^-methyladenosine and then used for cDNA construction and qPCR analysis. The primers used in m^6^A-qPCR assays are listed in Additional file 1: Table S2.

#### Statistical Analysis

For gene expression, statistical comparisons were performed by using one-way ANOVA as indicated in the figure legends. *P* < 0.05 was considered significant. For GO and KEGG analysis, the Benjamini and Hochberg method for false discovery rate (FDR-BH correction) was applied to correct for multiple comparisons. The number of biological (non-technical) replicates for each experiment was indicated in the figure legends.

## Results and Discussion

### Synthesis and Characterization of BSA-Templated NPs

**Fig. 2:**
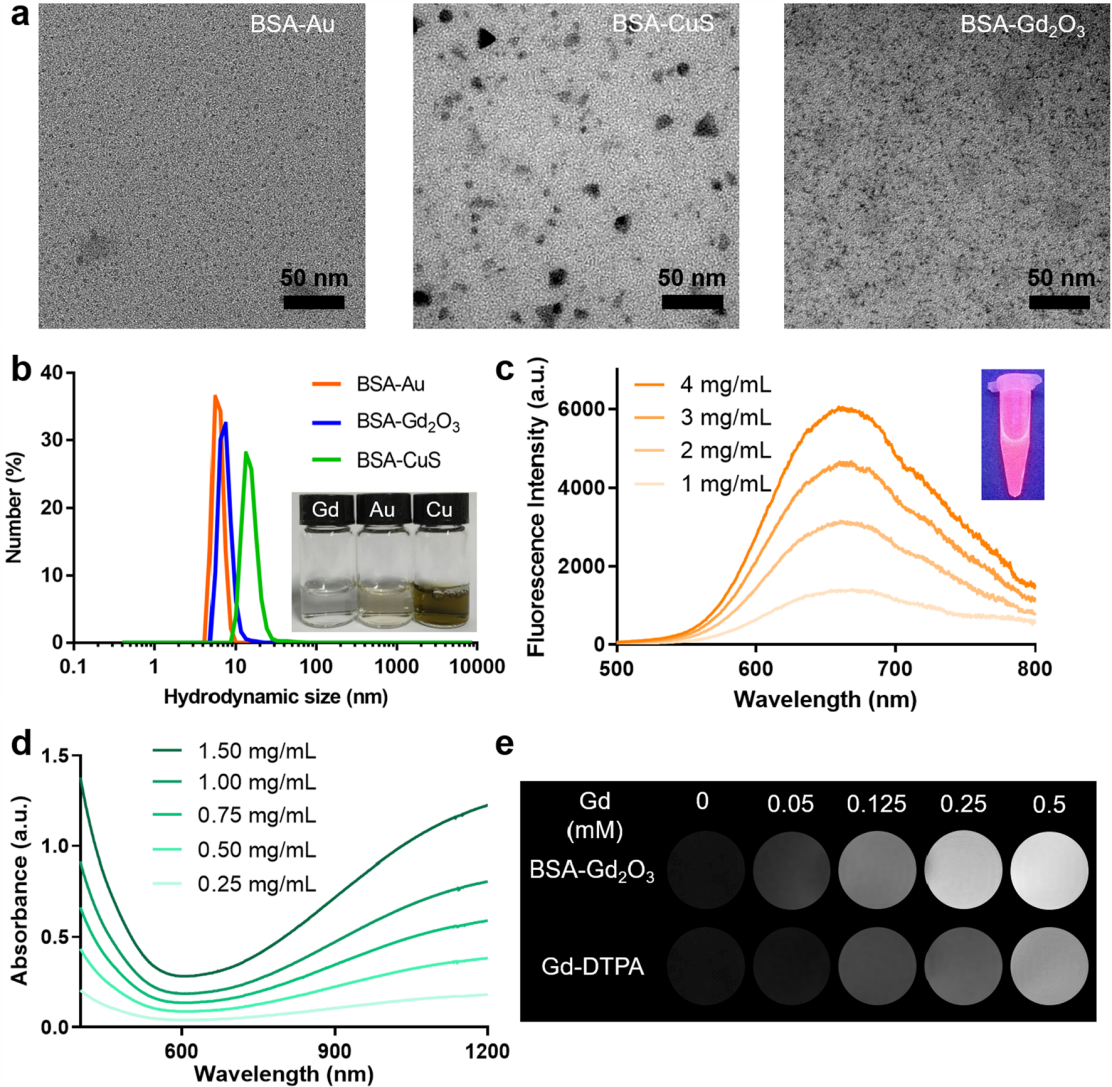
Characterization of BSA-templated NPs. **a** TEM images of BSA-Au, BSA-CuS, and BSA-Gd_2_O_3_ NPs. **b** Hydrodynamic sizes of BSA-Au, BSA-CuS, and BSA-Gd_2_O_3_ NPs. Inset: The photographs of obtained BSA-templated NPs dispersed in water (4 mg/mL). **c** Fluorescence spectra of BSA-Au NPs with different concentrations. Inset: The photographs of BSA-Au NPs solution (4 mg/mL) under ultraviolet light. **d** Absorption spectra of BSA-CuS NPs with different concentrations. **e** T_1_-weighted magnetic resonance images of BSA-Gd_2_O_3_ NPs and Gd-DTPA aqueous solutions with different concentrations of Gd

Three BSA-templated NPs were synthesized through a classical biomineralization method [53–55], in which BSA as a nanoreactor enables entrapping metal ions based on the interaction between functional groups (e.g., –SH, –NH_2_, and –COOH) and metal ions, and controlling the growth of NPs. These NPs showed ultra-small size in both TEM images (~ 3 nm for BSA- Au NPs, ~ 3 nm for BSA-Gd_2_O_3_ NPs, and ~ 10 nm for BSA-CuS NPs) (Fig. 2a) and hydrodynamic size measurement (5–20 nm) (Fig. 2b), which is benefited from BSA-directed controlled synthesis. BSA-Au NPs exhibited a strong fluorescent emission peaked at 664 nm (Fig. 2c), which can serve as an excellent fluorescent nanoprobe for biosensing and bioimaging [55]. The d–d transition of Cu^2+^ guarantees the strong near-infrared absorption of BSA-CuS NPs (Fig. 2d), making it a promising photothermal therapy agent for tumor ablation and antimicrobial treatment [53, 56]. BSA-Gd_2_O_3_ NPs owned a stronger magnetic resonance imaging capability than Gd-DTPA (Fig. 2e), and have been widely used for in vivo MR imaging [57, 58]. The characteristic FT-IR absorption bands of BSA confirmed the presence of BSA in these NPs (Additional file 1: Fig. S1), and all these NPs showed similar zeta potential with that of BSA solution, indicating BSA serves as the encapsulation layer (Additional file 1: Fig. S2). As shown in Additional file 1: Fig. S3, the appearance of both solid and solution of BSA-templated NPs exhibited no obvious changes, and no precipitate was observed in all solutions. The hydrodynamic sizes of these BSA-templated metallic nanoparticles did not change significantly (Additional file 1: Fig. S4). Consistently, the optical absorption spectra of BSA-CuS NPs (Additional file 1: Fig. S5), the fluorescence spectra of BSA-Au NPs (Additional file 1: Fig. S6), and the MR signal intensity of BSA-Gd_2_O_3_ NPs (Additional file 1: Fig. S7) did not change dramatically within 2 weeks no matter in solid or aqueous storage conditions. These results verified the good storage stability of BSA-templated metallic nanoparticles both in solid or aqueous conditions. We also preliminarily evaluated the protein corona formation in vitro by mixing the BSA-templated NPs with 10% FBS under 37 °C and monitoring the hydrodynamic sizes of solutions at different timepoints post-mixing. As shown in Additional file 1: Fig. S8, the hydrodynamic sizes of nanoparticles only and mixed solutions remained relatively stable (approximately 5–20 nm), which did not significantly alter as the incubation time increased. This indicated that BSA-templated NPs did not readily adsorb the proteins probably due to the own albumin template, which is consistent with previous report [59]. Despite the good storage stability and generally accepted biocompatibility of BSA-templated NPs [60–62], the potential effects of biomineralization NPs on the epitranscriptomic changes remain largely unknown.

### Global m^6^A Changes in HEK293T Cells Induced by BSA-Templated NPs

**Fig. 3:**
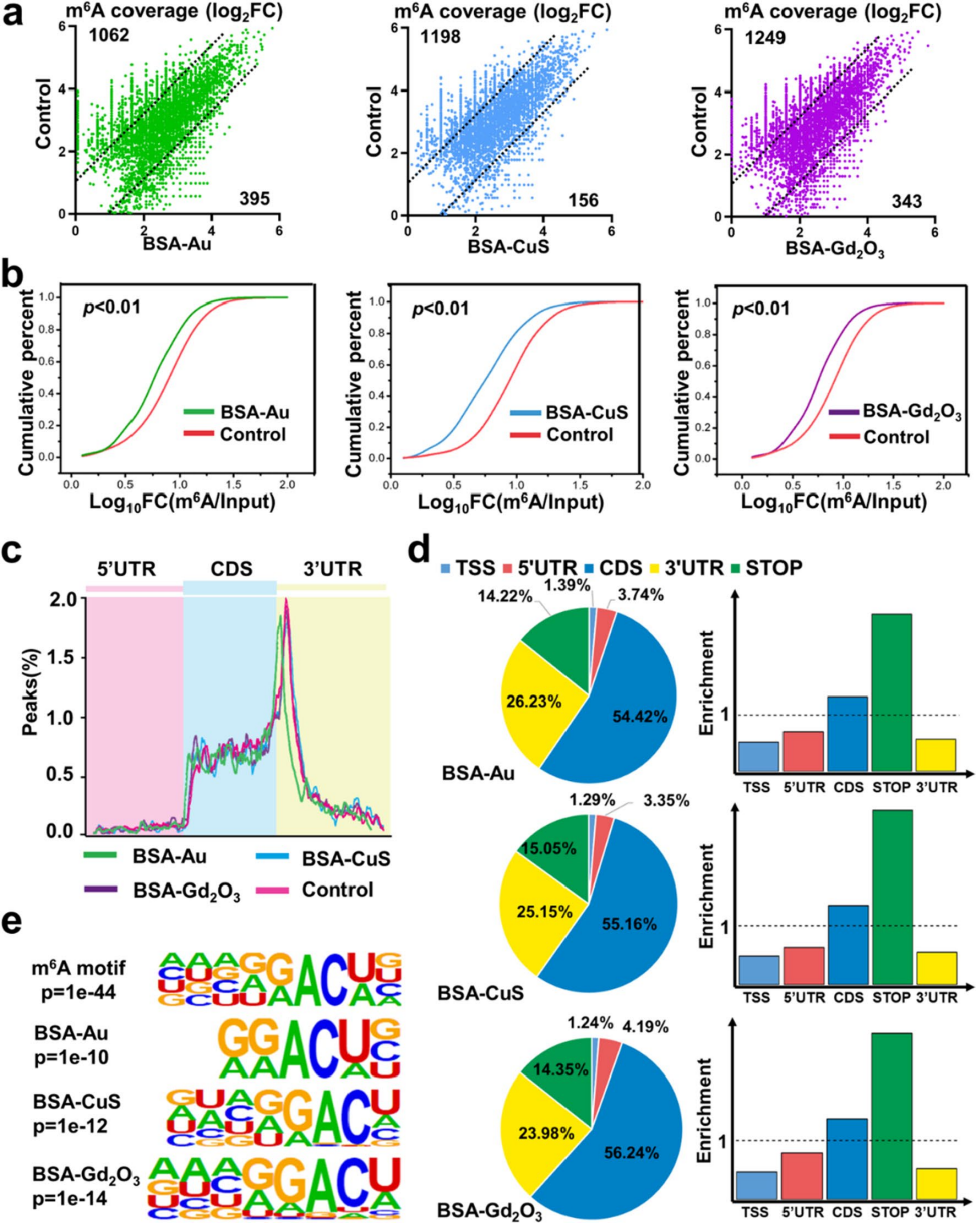
Global m^6^A changes induced by BSA-templated NPs. **a** Scatter plots showing the variation of m^6^A coverage of individual m^6^A sites in cells treated with BSA-templated NPs. Pair-wise comparison was shown between BSA-Au NPs (left), BSA-CuS NPs (middle), BSA-Gd_2_O_3_ NPs (right), and controls, respectively. The dashed lines indicated cut-off of log_2_FC(BSA-templated NPs/Control) (1 or −1). The numbers of genes with downregulated (log_2_FC < -1) or upregulated (log_2_FC > 1) m^6^A level was shown. FC, fold change. **b** Cumulative frequency of m^6^A targeted genes upon BSA-templated NPs exposure. P values were calculated using two-sided Wilcoxon and Mann–Whitney test. **c** Metagene profile of enrichment of m^6^A-targeted sites across mRNA transcriptome. 5’UTR, 5’ untranslated region; CDS, coding sequence; 3’UTR, 3’ untranslated region. **d** The distribution (left) and enrichment (right) of m^6^A peaks within different gene regions. Enrichment was determined by the proportion of m^6^A peaks normalized by the length of the region. **e** Top consensus sequences of m^6^A-targeted motif

In the assessment of cell viability upon the exposure of NPs, neither MTT analysis nor apoptosis assay exhibited obvious cell death when HEK293T cells were incubated with BSA-Au, BSA-CuS and BSA-Gd_2_O_3_ NPs at the concentrations of 50, 100, and 200 μg/mL (Additional file 1: Figs. S9 and S10). Similarly, no obvious cytotoxicity was observed on 3T3-L1 cells (a mouse embryonic fibroblastic cell line) when incubated with different BSA-templated NPs at different concentrations (Additional file 1: Fig. S11). We also quantitated the uptaken rates of BSA-Au, BSA-Gd_2_O_3_, and BSA-CuS NPs by HEK293T cells, which were 0.32%, 0.43%, and 2.11%, respectively (Additional file 1: Fig. S12). Then, the m^6^A-seq was performed to explore the NPs-induced RNA methylation changes. As shown in Fig. 3a, b, all the three NPs could induce both upregulated and downregulated m^6^A level. However, the number of genes with downregulated m^6^A level was much greater than that with upregulated m^6^A level, which led to the overall reduced m^6^A landscape. For example, BSA-CuS NPs induced downregulated m^6^A level in 1198 genes, which was far beyond that (156 genes) with upregulated m^6^A level. The genomic landscape of m^6^A distribution indicated that the m^6^A-binding sites of three NPs were similar (Fig. 3c, d). Most of the m^6^A-binding sites were located in protein-coding sequence and are highly enriched for stop codon and 3’UTR, which was consistent with the control group. Moreover, all the three NPs shared highly conserved m^6^A targets “GGACU” sequence (Fig. 3e), which matched well with the previously reported m^6^A consensus sequence “RRACH” (R = G or A, H = A, C or U) [63]. These findings indicated that BSA-templated NPs could reduce the global m^6^A landscape, but could not affect the distribution of m^6^A and consensus motif. In addition, it has been reported that m^6^A residues can be selectively recognized by the reader protein YTHDF2 to regulate the mRNA degradation [36]. Therefore, the significantly decreased m^6^A level hinted that BSA-templated NPs may own the ability to reprogram epitranscriptome for stability regulation of targeted mRNA.

### Functional Annotation of Genes with NPs-Induced m^6^A Changes

**Fig. 4:**
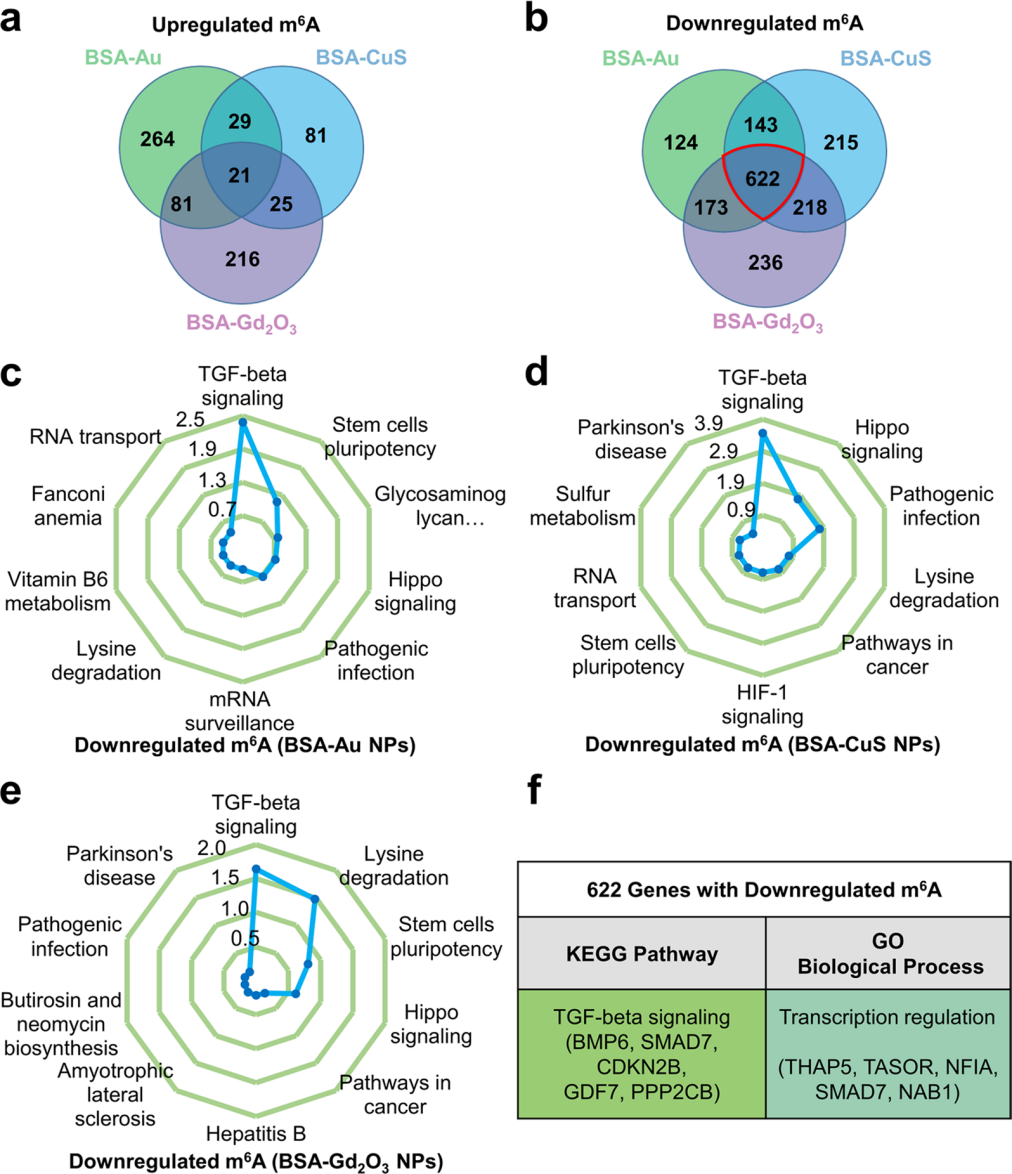
Functional annotation of the genes with NPs-induced m^6^A changes. **a** Venn diagram of genes with upregulated m^6^A treated with various NPs. **b** Venn diagram of genes with downregulated m^6^A treated with various NPs. **c–e** KEGG pathway analysis of genes with downregulated m^6^A level treated with different NPs. The axis refers to the -log_10_FDR(*p* value). **f** The most prominent pathway and biological process from the KEGG and GO analysis of three NPs-shared 622 genes with downregulated m^6^A level, respectively

Given the important role of m^6^A in various biological processes, the biological functions of genes with NPs-induced m^6^A changes were further investigated by enrichment analysis. As shown in Fig. 4a, b, the cells treated with BSA-Au, BSA-CuS and BSA-Gd_2_O_3_ NPs displayed both some overlapped peaks (genes with m^6^A) and unique ones no matter with upregulated or downregulated m^6^A level. Considering that different m^6^A peak patterns are related to different cellular functions, functional modeling for genes with upregulated or downregulated m^6^A level under treatment of different BSA-templated NPs was respectively conducted with Kyoto Encyclopedia of Genes and Genomes (KEGG) and gene ontology (GO) analysis. The numbers of genes with downregulated m^6^A were 1062, 1198 and 1249 under treatment of BSA-Au, BSA-CuS and BSA-Gd_2_O_3_ NPs, respectively. In the KEGG pathway analysis, these genes with downregulated m^6^A level were enriched in multiple pathways, but all pointed to the TGF-beta signaling (Fig. 4c–e). In the annotation of GO, the genes with downregulated m^6^A level were enriched in diverse biological processes, such as RNA metabolic process, nucleic acid process and transcription regulation (Additional file 1: Fig. S13). The differences among BSA-Au, BSA-CuS, and BSA-Gd_2_O_3_ NPs may be attributed to their distinct physicochemical properties and released metal ions. In spite of this, some common pathways and biological processes could be found among the three NPs-treated HEK293T cells. Particularly, there were 622 common genes with downregulated m^6^A level among the three NPs-treated cells (Fig. 4b). The KEGG analysis of the 622 genes showed that TGF-beta signaling was the most enriched pathway, which was associated with multiple genes like BMP6, SMAD7, CDKN2B, GDF7, and PPP2CB (Fig. 4f). In the meantime, the transcription regulation was the most relevant process as indicated by the GO analysis of the 622 common genes (Fig. 4f). Then, we used MeRIP-qPCR to quantify the m^6^A level of representative genes associated with TGF-beta signaling and transcription regulation process. The results showed that the m^6^A level of BMP6, CDKN2B, GDF7, PPP2CB, TASOR and NAB1 were attenuated by BSA-templated NPs with different patterns (Additional file 1: Figs. S14 and S15), which was consistent with m^6^A-seq results. It is worth mentioning that TGF-beta signaling plays vital roles in a diverse set of cellular processes, such as cell proliferation, recognition, differentiation, apoptosis, and specification of developmental fate [64]. The KEGG and GO analysis of genes with upregulated m^6^A level induced by the NPs were also displayed (Additional file 1: Figs. S16 and S17). These functional annotations suggested that the genes with m^6^A variations induced by BSA-templated NPs were related to multiple pathways and biological processes, particularly the genes with reduced m^6^A level were enriched for TGF-beta signaling.

### Potential Reasons for m^6^A Variations Induced by the NPs

**Fig. 5:**
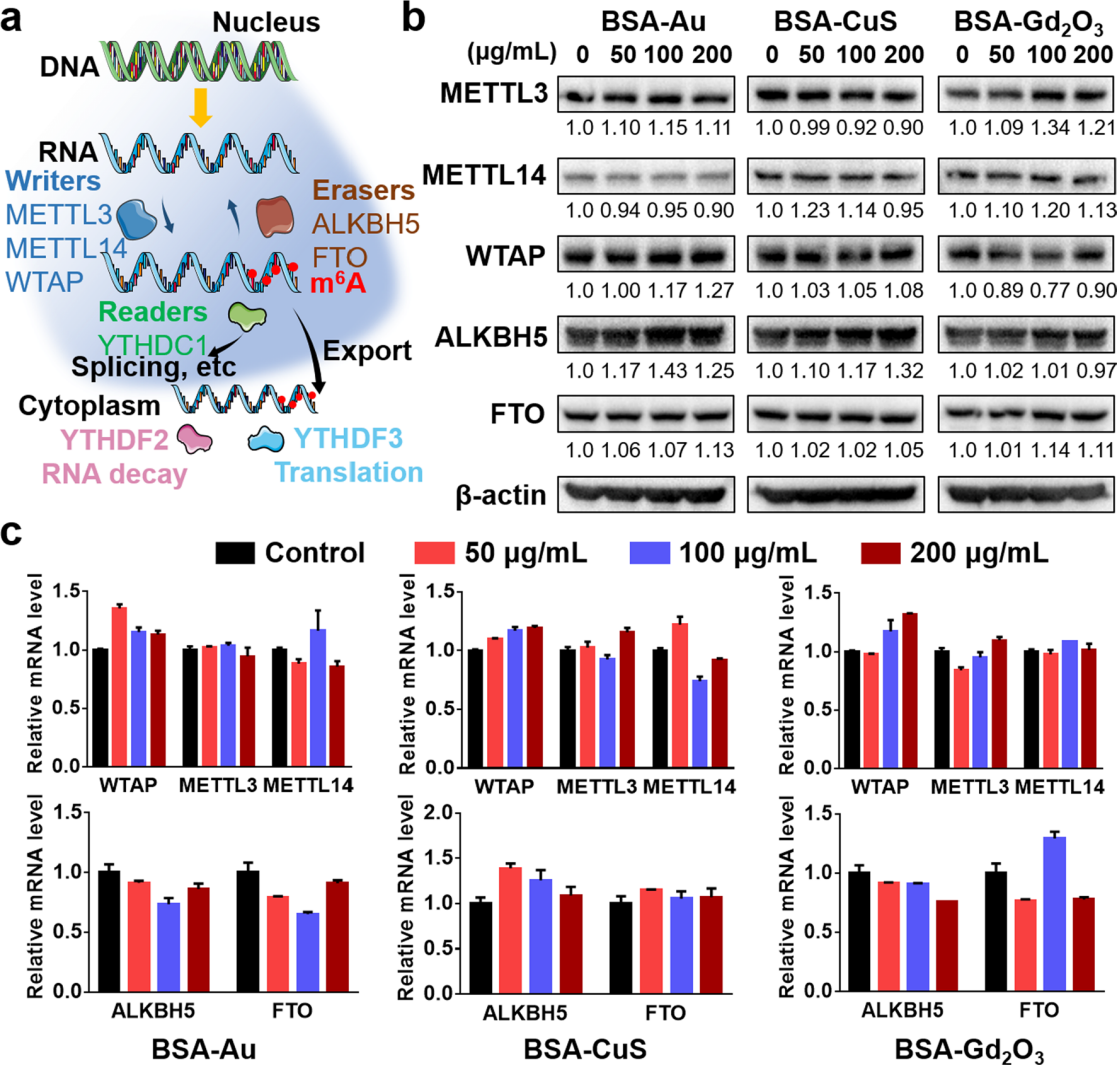
Expression changes of m^6^A-related writers and erasers upon BSA-templated NPs exposure. **a** Schematic representation of the m^6^A manipulation and function. **b** Western blot analysis of m^6^A-related protein expression in HEK293T cells upon BSA-templated NPs treatment for 24 h. Left panel: exposure to BSA-Au NPs, middle panel: exposure to BSA-CuS NPs, right panel: exposure to BSA-Gd_2_O_3_ NPs. A total of 30 μg protein was loaded (β-actin was used as a control for loading). **c** Relative mRNA levels of m^6^A-related genes in HEK293T cells upon NPs exposure for 24 h. Left panel: exposure to BSA-Au NPs; middle panel: exposure to BSA-CuS NPs; right panel: exposure to BSA-Gd_2_O_3_ NPs. Data were shown as the mean ± SD

The m^6^A mRNA methylation is regulated and exerts its functions by three groups of “m^6^A RNA modifiers” including m^6^A methyltransferases (writers), m^6^A demethylases (erasers), and m^6^A binding proteins (readers) (Fig. 5a). The writers (e.g., METTL3, METTL14 and WTAP) facilitate the synthesis of m^6^A [65, 66], the erasers (e.g., ALKBH5 and FTO) catalyze the demethylation of m^6^A [67, 68], and the readers (e.g., YTHDC1, YTHDF2 and YTHDF3) specifically recognize m^6^A and regulate its functions, such as splicing and translation [63, 69]. According to the functions of writers, erasers and readers, we hypothesized that the m^6^A alteration induced by BSA-templated NPs was resulted from the dysregulation of the m^6^A-related writers and erasers. To test the hypothesis, we measured gene expression and protein levels of these modifiers in HEK293T cells after being exposed to BSA-templated NPs. As shown in Fig. 5b, c and Additional file 1: S18, these genes did not exhibit a significantly regular expression pattern after exposure to these NPs. This result indicated that the BSA-templated NPs may not induce the m^6^A variations by directly affecting the expression level of m^6^A-related enzymes, and possible interpretations need to be further explored.

**Fig. 6:**
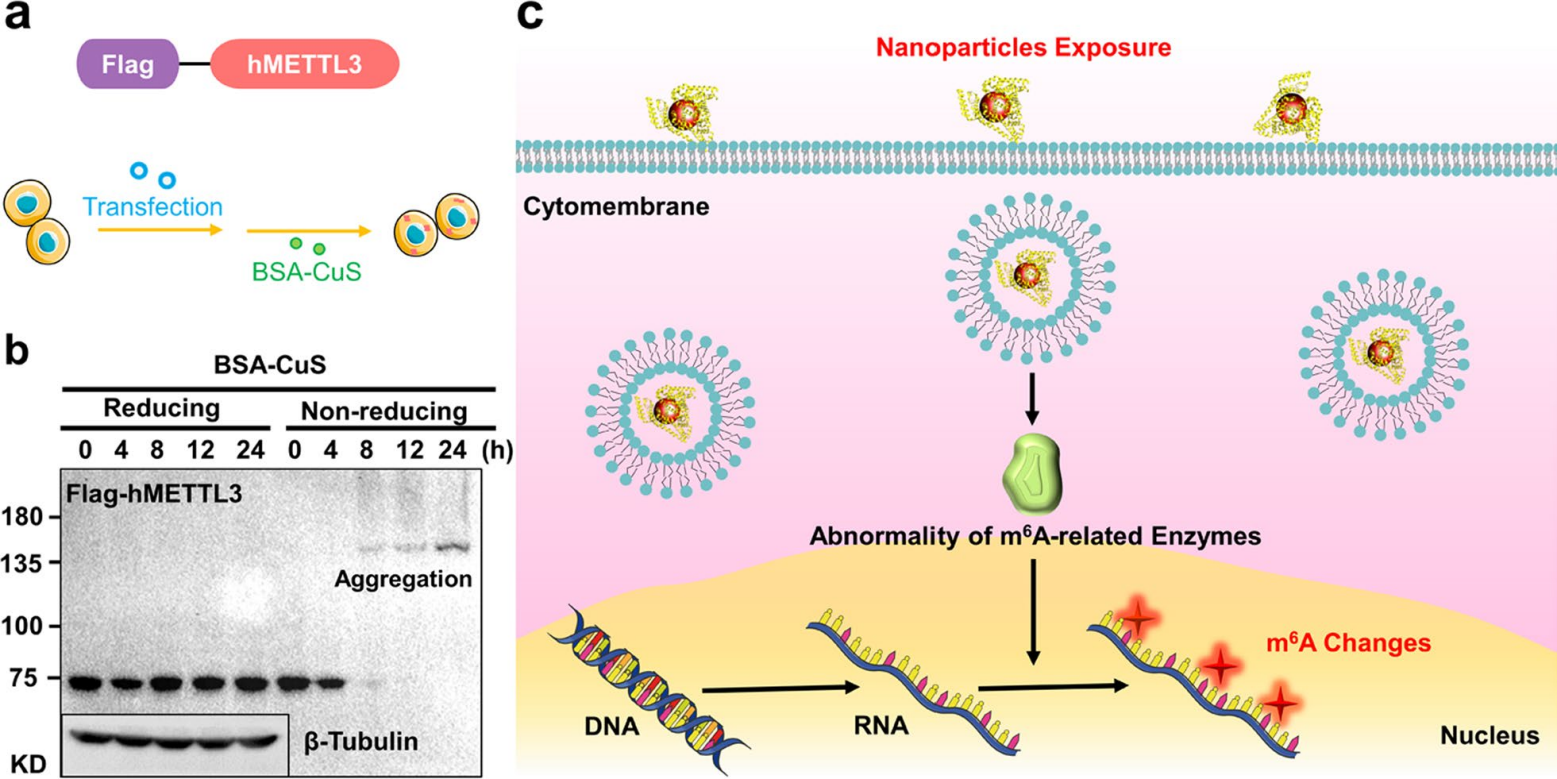
Possible mechanism for m^6^A changes induced by the BSA-templated NPs. **a** Schematic illustration of transfection of Flag-tagged human METTL3 (Flag-hMETTL3) into HEK293T cells, followed by exposure to 200 μg/mL BSA-CuS NPs for 24 h. **b** Redox-western blot analysis of Flag-hMETTL3 in reduced and non-reduced conditions. A total of 50 μg protein was loaded (β-tubulin was used as a control for loading). **c** Schematic representation of the potential mechanism for m^6^A changes induced by the BSA-templated NPs

It has been found that glutathione-based nanoclusters can cause Ten-Eleven-Translocation (TET) proteins aggregation, which affects DNA methylation and hydroxyl-methylation [49]. This inspires us to suppose that if NPs could induce abnormal aggregation of m^6^A-related enzymes, leading to the m^6^A distribution reconstruction. To verify the hypothesis, the writer METTL3, one of the earliest discovered m^6^A-related enzymes but with no significant expression change in previous assays [70], was taken into investigation. Flag-tagged METTL3 was transfected into HEK293T cells before exposure to BSA-CuS NPs, which induced the most significant m^6^A change (Fig. 6a). As shown in Fig. 5b, BSA-CuS NPs (200 μg/mL) did not induce significant change in METTL3 expression within 24 h-exposure. Next, to validate if BSA-CuS NPs could affect METTL3, a redox-western blot assay was performed. METTL3 protein complex could be observed under non-reducing condition (Fig. 6b), and more complex formed as incubation time extended. This suggested that BSA-CuS NPs could induce abnormal aggregation of m^6^A writer METTL3. METTL3 is the catalytic subunit in m^6^A methyltransferase complex that transfers a methyl group from S-adenosylmethionine (SAM) to an adenosine in RNA, and the abnormal structure of METTL3 may directly affect its enzymatic activity for transmethylation. Thus, the aggregation of m^6^A writer METTL3 induced by the NPs may be another underlying mechanism for reprogramming m^6^A enrichment and epitranscriptome.

Based on the above results, despite no obviously detected apoptosis in cells upon exposure of BSA-templated NPs, these NPs could indeed induce the m^6^A changes and potentially affect the cellular biological functions, showing an unprecedented epitranscriptomic scenery induced by NPs. In addition, the potential mechanism for m^6^A changes induced by the BSA-templated NPs has been preliminarily revealed. As shown in Fig. 6c, BSA-templated NPs could result in the abnormality of m^6^A-related enzymes, such as aggregation of m^6^A writers, which at least partly contributed to the alterations of m^6^A landscape.

## Conclusions

In this proof-of-concept study, we studied the epitranscriptomic impact (m^6^A) of biomineralization nanoparticles and explored its potential biological mechanisms. We found that BSA-templated NPs could induce epitranscriptomic abnormalities (e.g., reduced m^6^A level), which cannot be detected by conventional biotoxicity assessments. The possible mechanism could be at least partly deduced that the BSA-templated NPs may induce the aggregation of m^6^A-related enzymes to affect the m^6^A distribution. However, the underlying mechanisms of how NPs affect these m^6^A-related enzymes still need further investigation in the future. Taken together, epitranscriptomics analysis could provide an unprecedented finding of the biological effect induced by NPs, which should be integrated into the nanotoxicity evaluation systems for nanomaterials for their potential clinical translation.

## Supplementary Information

**Additional file 1**. Sequences of PCR primers; FT-IR spectra, Zeta potential, hydrodynamic sizes, absorption spectra, fluorescence spectra, and magnetic resonance imaging of NPs; cell viabilities; cellular uptaken rates of NPs; GO and KEGG analysis; PCR analysis, and Western blot analysis.

## Data Availability

All data supporting the conclusions of this article are included within the article.

## Abbreviations

m^6^A: N6-methyladenosine
BSA: Bovine serum albumin
AOP: Adverse outcome pathway
QSAR: Quantitative structure–activity relationship
NPs: Nanoparticles
TEM: Transmission electron microscopy
FT-IR: Fourier transform infrared
MTT: Methyl Thiazolyl Tetrazolium
cDNA: Complementary DNA
