# Supplementary material for: RNA m6A Alterations Induced by Biomineralization Nanoparticles: A Proof-of-Concept Study of Epitranscriptomics for Nanotoxicity Evaluation
Source: Nanoscale Res Lett. 2022 Feb 5;17:23. doi: 10.1186/s11671-022-03663-x (PMC8817964; doi:10.1186/s11671-022-03663-x)
Supplement: Supplementary file 1 — Additional file 1. Sequences of PCR primers; FT-IR spectra, Zeta potential, hydrodynamic sizes, absorption spectra, fluorescence spectra, and magnetic resonance imaging of NPs; cell viabilities; cellular uptaken rates of NPs; GO and KEGG analysis; PCR analysis, and Western blot analysis. [file 11671_2022_3663_MOESM1_ESM.docx]

Supplementary Information

RNA m^6^A Alterations Induced by Biomineralization Nanoparticles: A Proof-of-Concept Study of Epitranscriptomics for Nanotoxicity Evaluation

Jinbin Pan^1,†,^*, Jiaojiao Wang^3,†^, Kun Fang^4,†^, Wenjing Hou^5^, Bing Li^2^, Jie Zhao^2,^*, Xinlong Ma^2,^*

^1^ Department of Radiology, Tianjin Key Laboratory of Functional Imaging, Tianjin Medical University General Hospital, Tianjin 300052, China

^2^ Department of Orthopedics, Tianjin Hospital, Tianjin University, Tianjin 300211, China

^3^ Department of Radiology, Second Hospital of Tianjin Medical University, Tianjin 300211, China

^4^ Department of Radiology, Beijing Chest Hospital, Capital Medical University, Beijing Tuberculosis and Thoracic Tumor Research Institute, Beijing 101149, China

^5^ Department of Diagnostic and Therapeutic Ultrasonography, Tianjin Medical University Cancer Institute and Hospital, National Clinical Research Center of Cancer, Key Laboratory of Cancer Prevention and Therapy, Tianjin's Clinical Research Center for Cancer, Tianjin 300060, China

^†^ These authors contributed equally to this work.

^*^ Corresponding author (e-mail: panjinbin@ tmu.edu.cn; zhaojie@tmu.edu.cn; maxinlong8686@yeah.net)

**Table S1** Real-time PCR primers

|  | Forwad primer | Reverse primer |
| --- | --- | --- |
| hMETTL3^1^ | TTGTCTCCAACCTTCCGTAGT | CCAGATCAGAGAGGTGGTGTAG |
| hMETTL14 | AGTGCCGACAGCATTGGTG | GGAGCAGAGGTATCATAGGAAGC |
| hFTO | ACTTGGCTCCCTTATCTGACC | TGTGCAGTGTGAGAAAGGCTT |
| hWTAP | CTTCCCAAGAAGGTTCGATTGA | TCAGACTCTCTTAGGCCAGTTAC |
| hALKBH5 | CGGCGAAGGCTACACTTACG | CCACCAGCTTTTGGATCACCA |
| hYTHDC1 | AACTGGTTTCTAAGCCACTGAGC | GGAGGCACTACTTGATAGACGA |
| hYTHDF2 | CCTTAGGTGGAGCCATGATTG | TCTGTGCTACCCAACTTCAGT |
| hYTHDF3 | TCAGAGTAACAGCTATCCACCA | GGTTGTCAGATATGGCATAGGCT |
| hACTB  hGAPDH | CATGTACGTTGCTATCCAGGC  GGAGCGAGATCCCTCCAAAAT | CTCCTTAATGTCACGCACGAT  GGCTGTTGTCATACTTCTCATGG |

^1)^ h (human)

**Table S2** Sequences of MeRIP-qPCR primers

|  | Forwad primer | | Reverse primer |
| --- | --- | --- | --- |
| BMP6-m^6^A | | TGCCATCTCGGTTCTTTACTTTG | GTGCTTCCGTGTTTTTTTAAGGCA |
| SMAD7-m^6^A | | GCAGATCAGCTTTGTGAAGGGCT | TTGCATGAAAAGCAAGCACTCAGG |
| CDKN2B-m^6^A | | ACCGCGACGTTGCAGGGTACCT | GGGTGGGGGTGGGAAATTGGGTA |
| GDF7-m^6^A | | CGCAAGCCGTTGCACGTGGACTT | CTCGAGGTGCGAACGCAAAGGGA |
| PPP2CB-m^6^A | | CAGATCCAGATGATCGTGGTGGA | TCCATTACAAGCTGGTGGGCACG |
| THAP5-m^6^A | | CCTTGTATAAGGATGTAGACTATGG | GCTCTTTTAACTCTAGAAGAGTTATC |
| TASOR -m^6^A | | CTTGTTCTTGTTAAACCTTTGGG | GGTTGAACCTTTTTGTGAAGTAAC |
| NAB1 -m^6^A | | AGGAGTTTTTGGAAATCATGGCAC | AGATGGGTATGCTACTGACAGGAA |
| NFIA -m^6^A | | GCCTTGTAAAGTCCCCACAATGC | GGCTGGCTGGGACTTTCAGATTG |


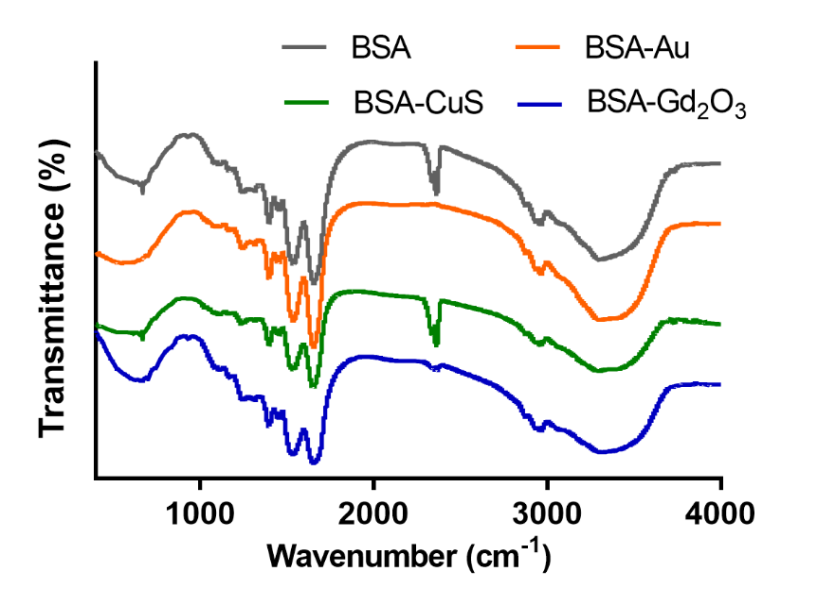


**Fig. S1** FT-IR spectra of BSA, BSA-Au, BSA-CuS, and BSA-Gd_2_O_3_ NPs.

**Fig. S2** Zeta potential of BSA, BSA-Au, BSA-CuS, and BSA-Gd_2_O_3_ NPs.


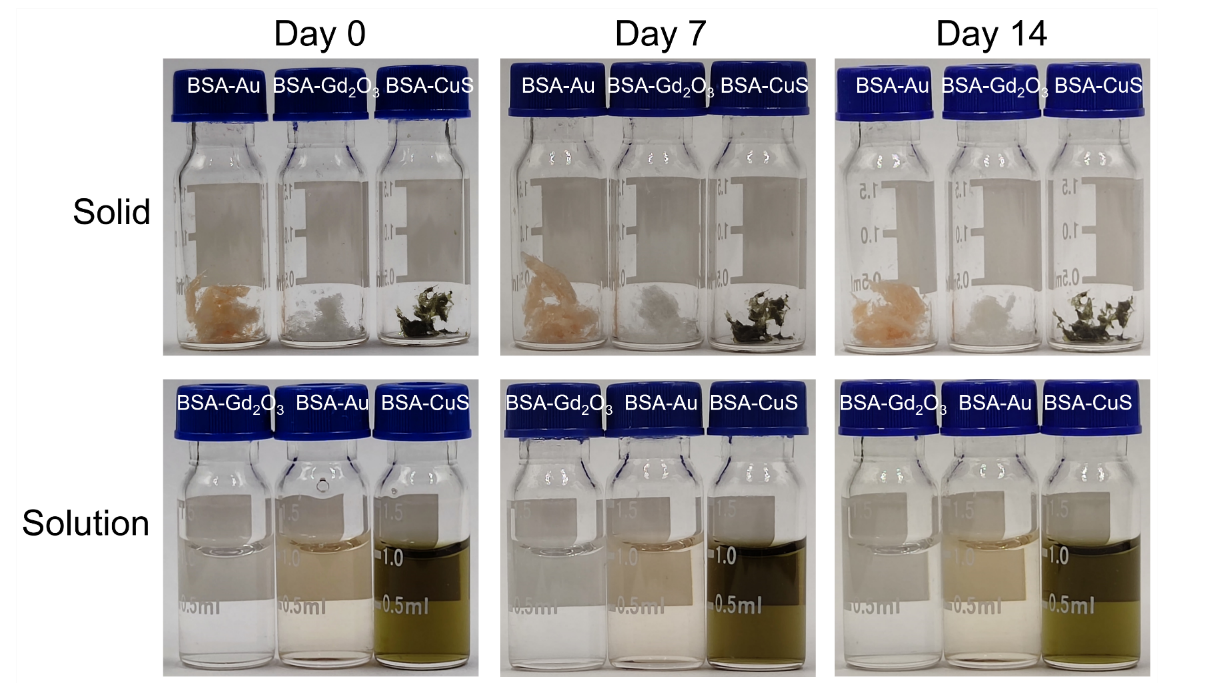


**Fig. S3** Common state of both solid and solution (4 mg/mL) of BSA-templated metallic nanoparticles.


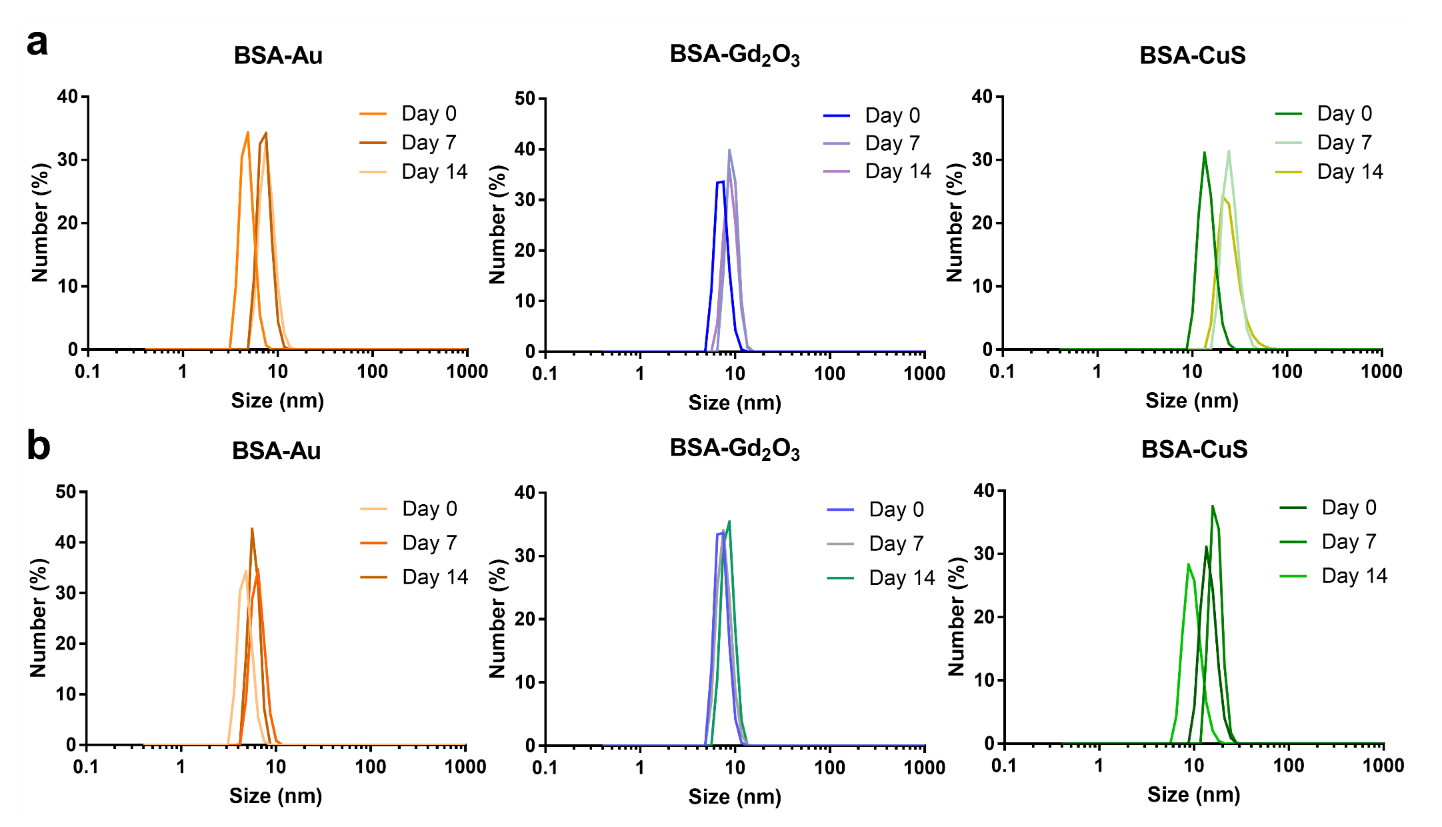


**Fig. S4** Hydrodynamic sizes of BSA-templated metallic nanoparticles within 2 weeks in aqueous (a) and solid (b) storage conditions.


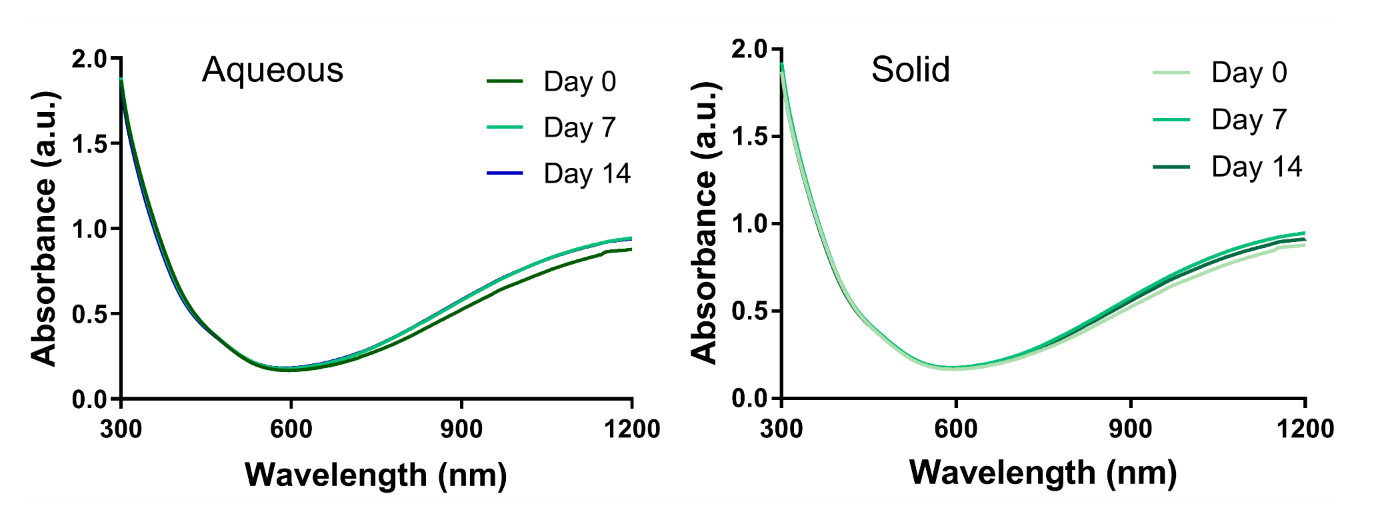


**Fig. S5** Absorption spectra of BSA-CuS NPs (1 mg/mL) within 2 weeks in aqueous and solid storage conditions.


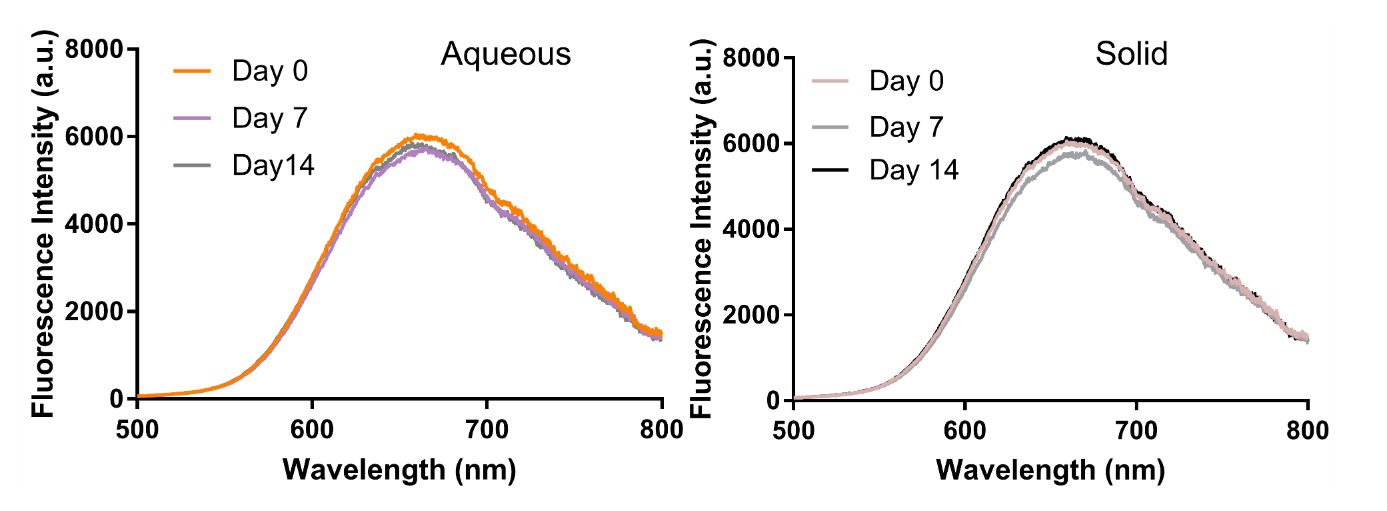


**Fig. S6** Fluorescence spectra of BSA-Au NPs (4 mg/mL) within 2 weeks in aqueous and solid storage conditions.


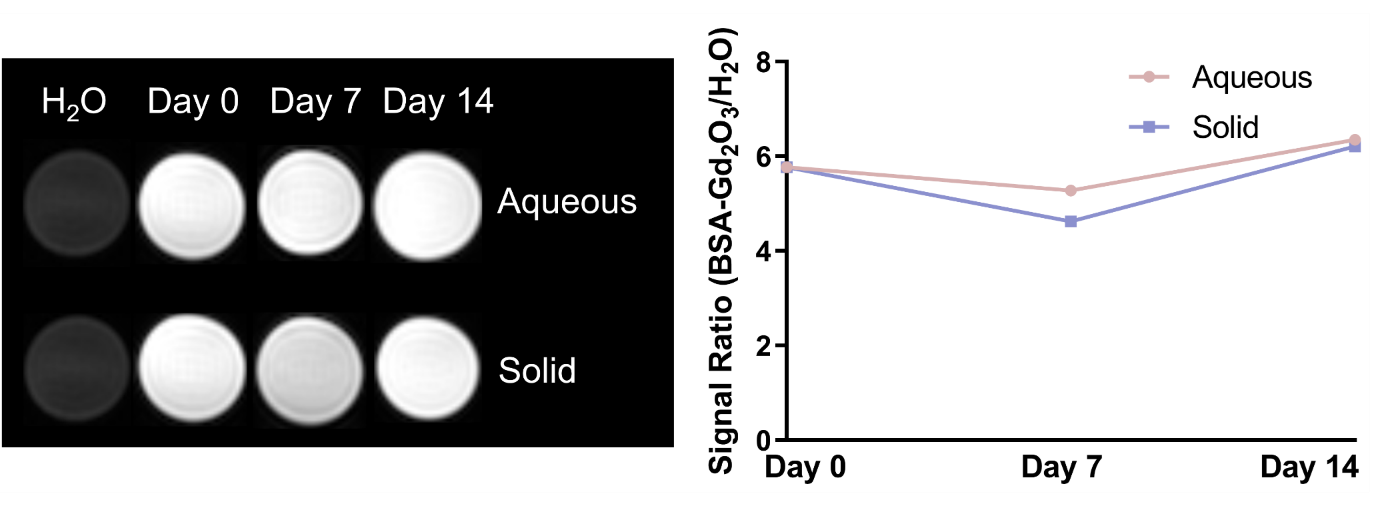


**Fig. S7** T1-weighted magnetic resonance images and intensity of BSA-Gd_2_O_3_ NPs (4 mg/mL) within 2 weeks in aqueous and solid storage conditions.


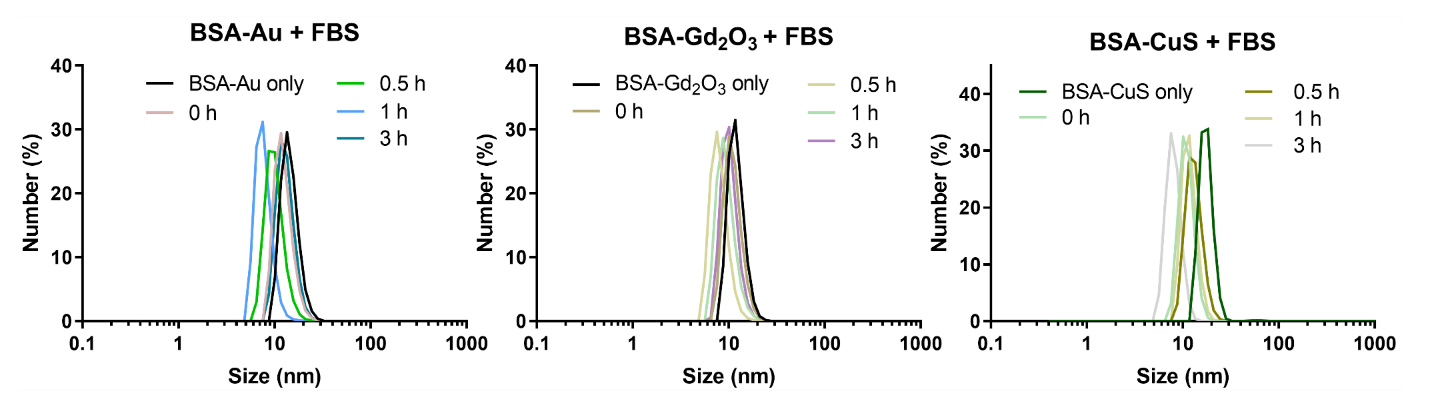


**Fig. S8** Hydrodynamic sizes of the mixed solutions of BSA-templated metallic nanoparticles and 10% FBS at different timepoints (0, 0.5, 1, and 3 h) post mixing.


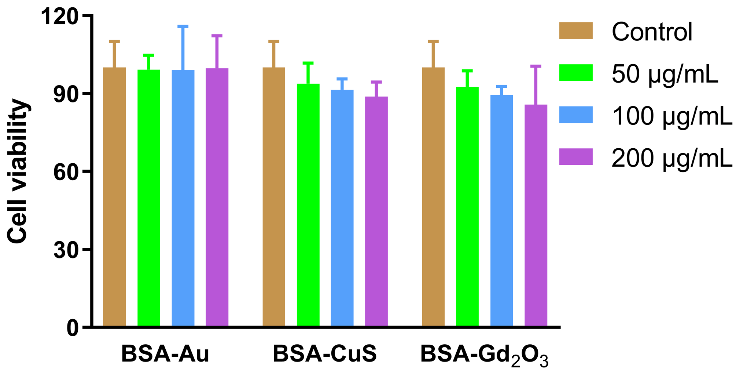


**Fig. S9** Cell viabilities of HEK293T cells upon exposure of BSA-templated NPs with different concentrations.


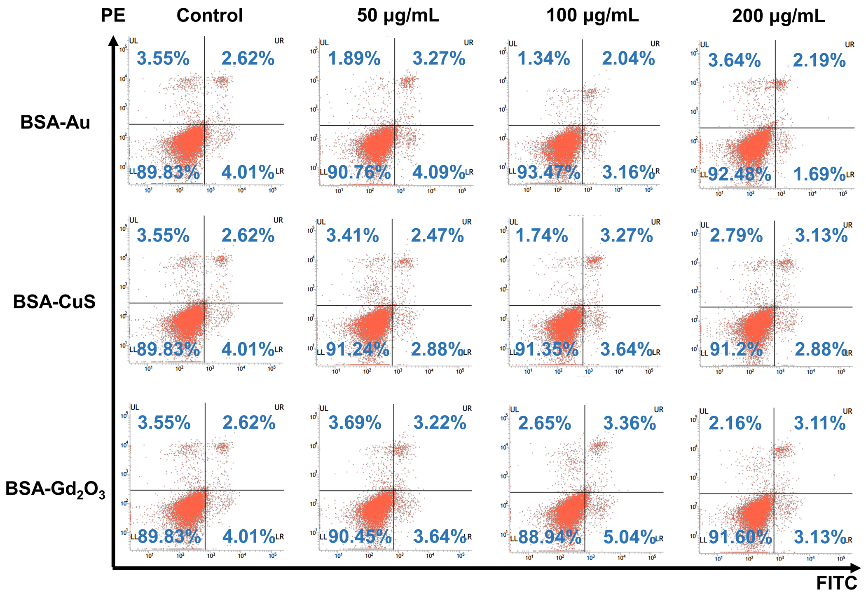


**Fig. S10** Cell apoptosis of HEK293T cells upon exposure of BSA-templated NPs with different concentrations.


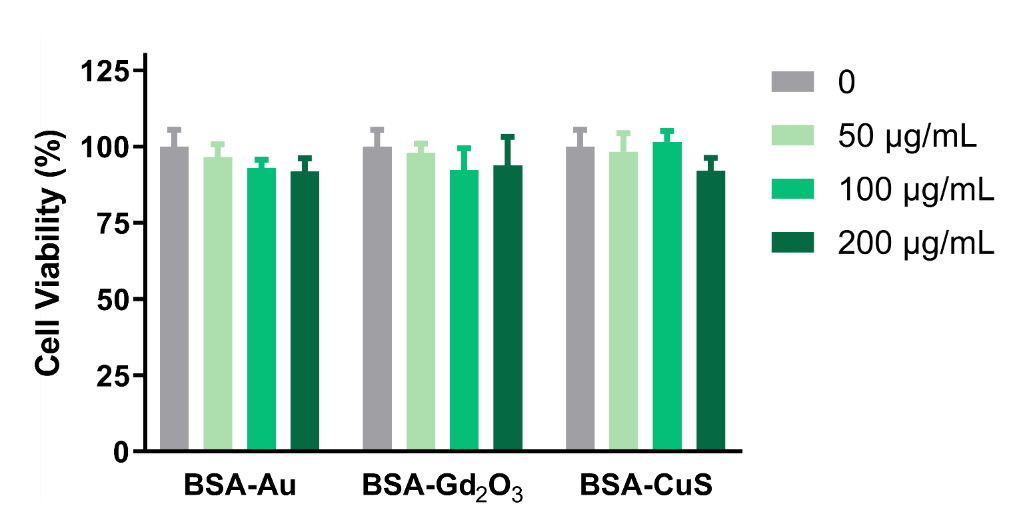


**Fig. S11** Cell viabilities of 3T3-L1 cells upon exposure to BSA-templated NPs with different concentrations.


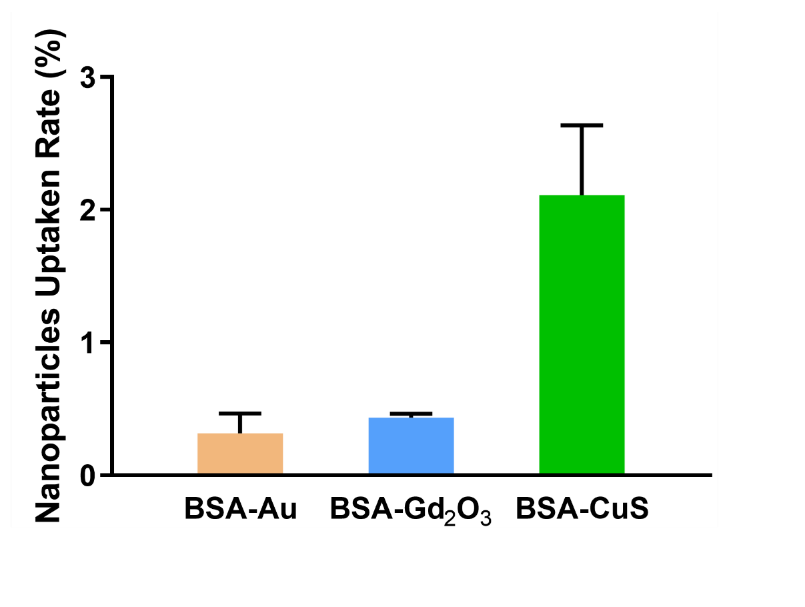


**Fig. S12** Cellular uptaken rates of BSA-templated NPs (200 μg/mL) by HEK293T cells.


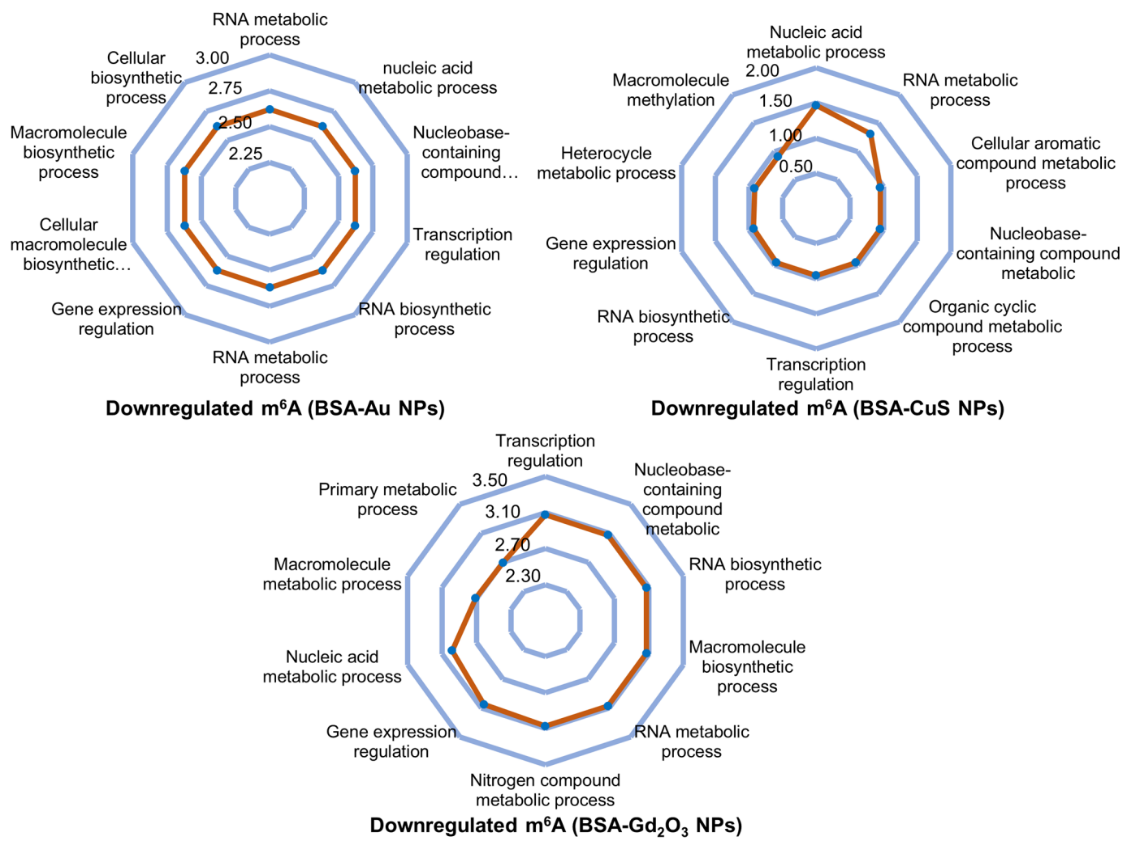


**Fig. S13** GO biological process analysis of genes with downregulated m^6^A level in HEK293T cells treated with NPs. The axis refers to the -log_10_FDR(p value).

**
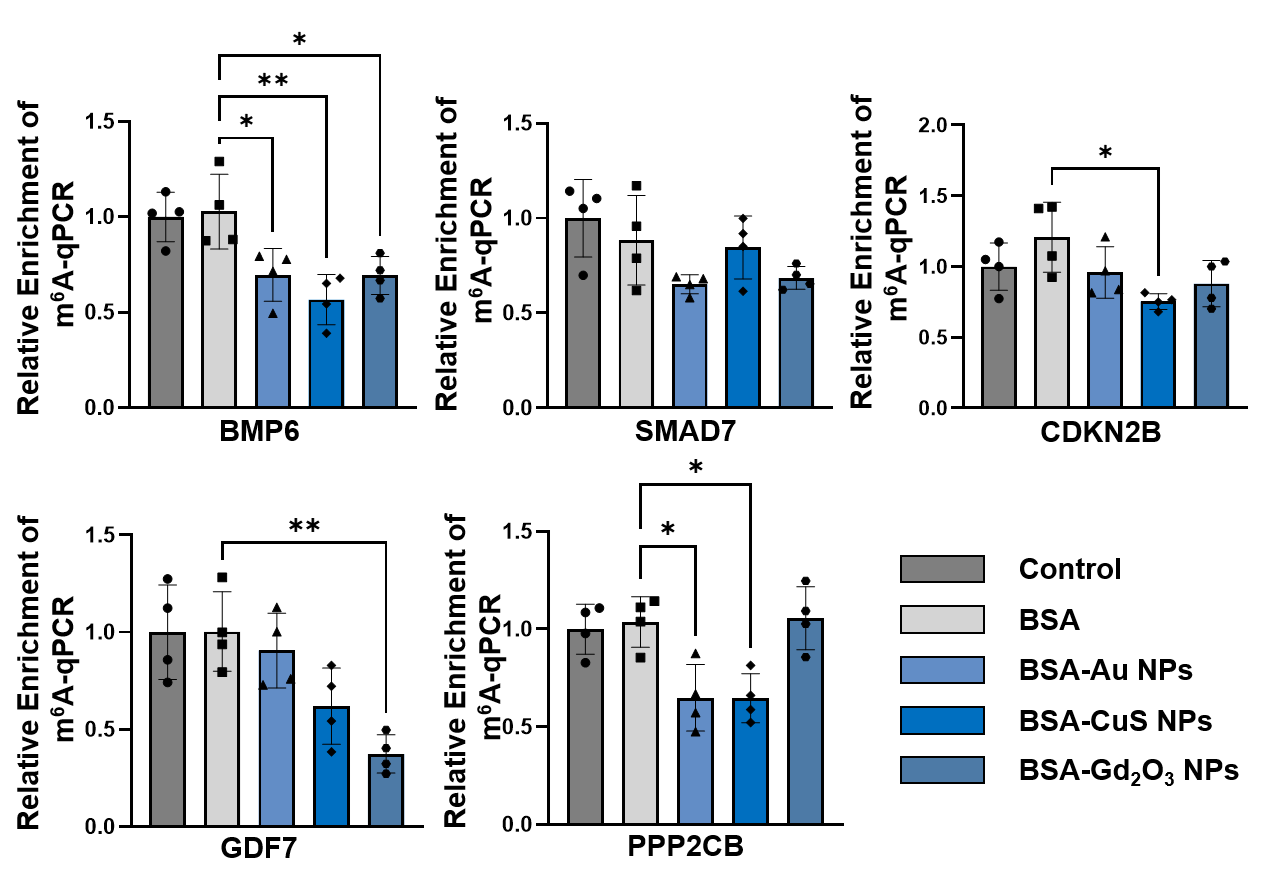
**

**Fig. S14** MeRIP-qPCR analysis of m^6^A levels of representative TGF-β signaling associated genes in HEK293T cells exposed to BSA-templated NPs. Error bars, mean ± SEM, *p < 0.05, **p < 0.01, n = 4 independent experiments.


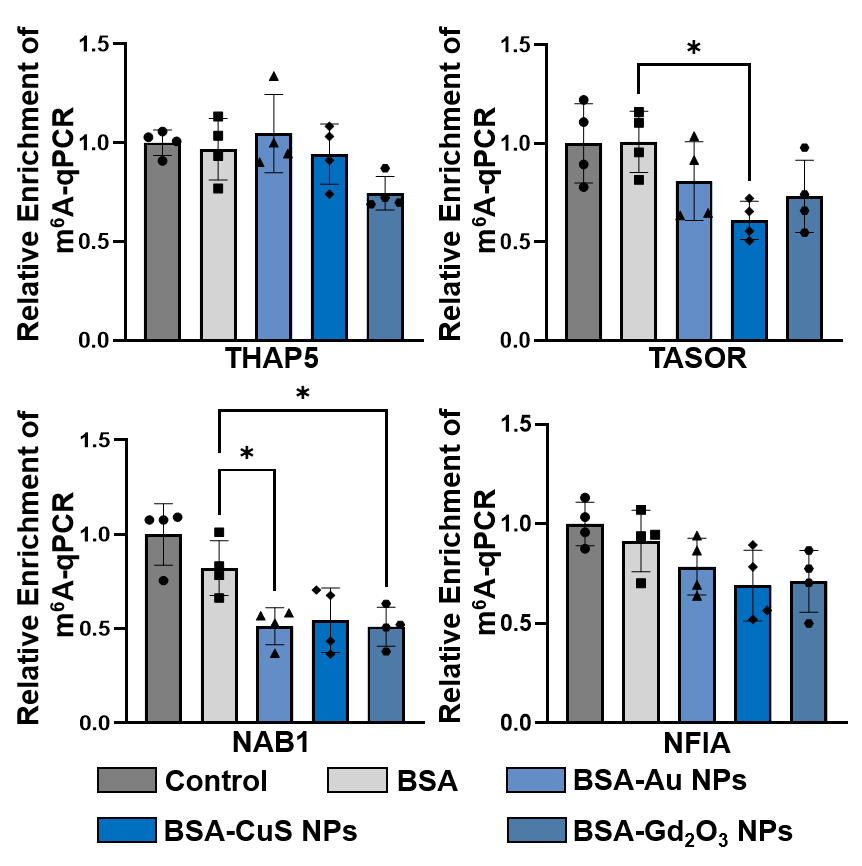


**Fig. S15** MeRIP-qPCR analysis of m^6^A levels of representative transcription regulation process associated genes in HEK293T cells exposed to BSA-templated NPs. Error bars, mean ± SEM, *p < 0.05, **p < 0.01, n = 4 independent experiments.


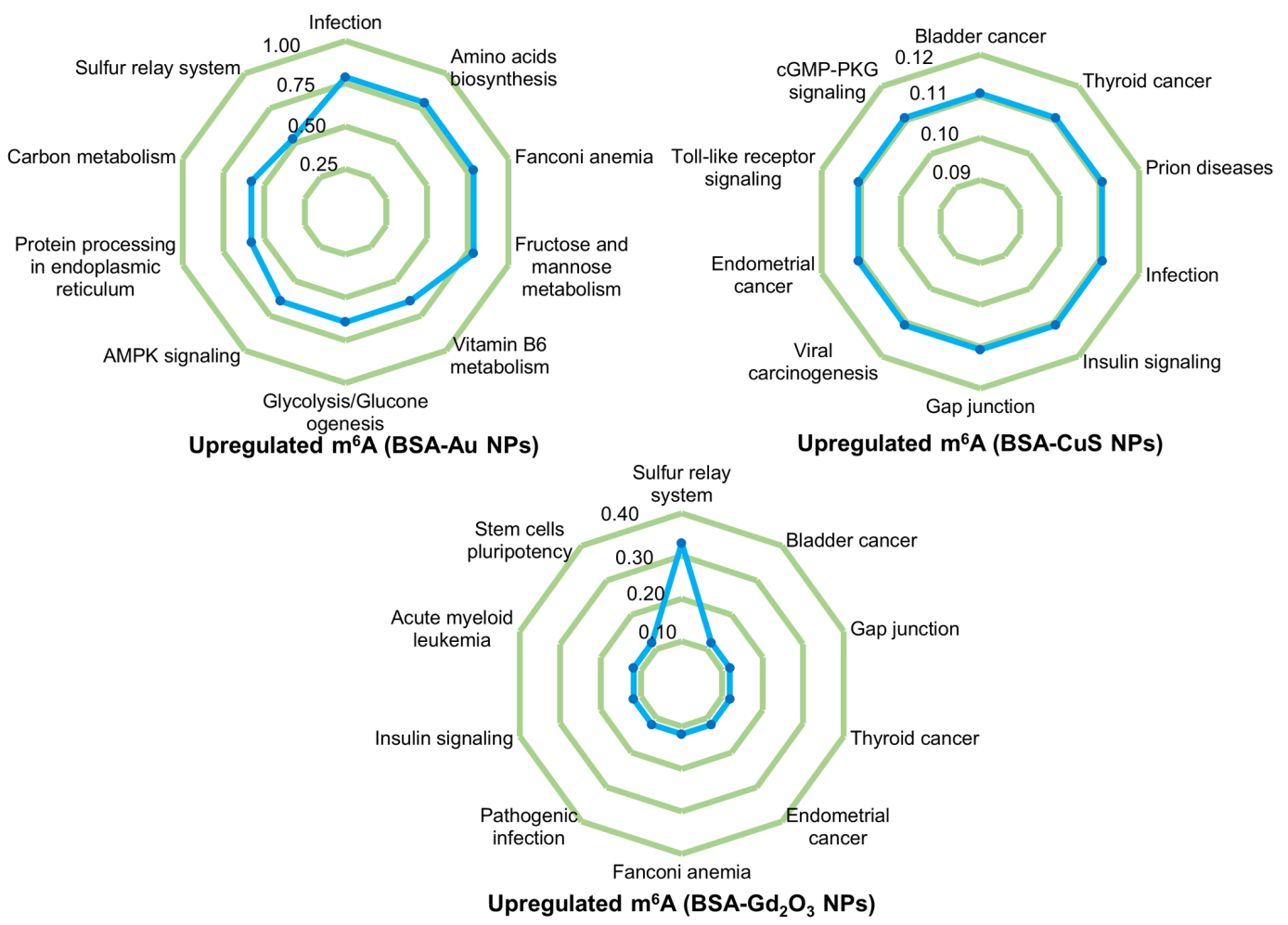


**Fig. S16** KEGG pathway analysis of genes with upregulated m^6^A level in HEK293T cells treated with NPs. The axis refers to the -log_10_FDR(p value).


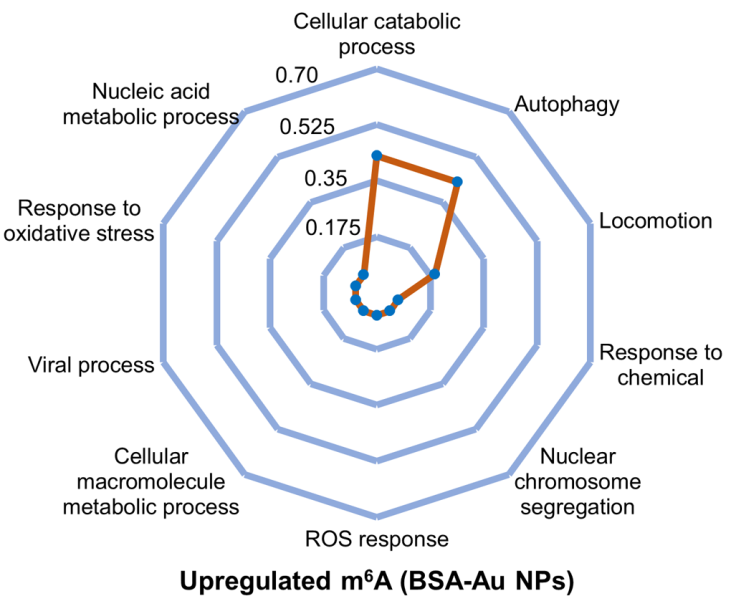


**Fig. S17** GO biological process analysis of genes with upregulated m^6^A level in HEK293T cells treated with BSA-Au NPs. The genes with upregulated m^6^A level in HEK293T cells treated with BSA-CuS and BSA-Gd_2_O_3_ NPs were insufficient for GO analysis. The axis refers to the -log_10_FDR(p value).


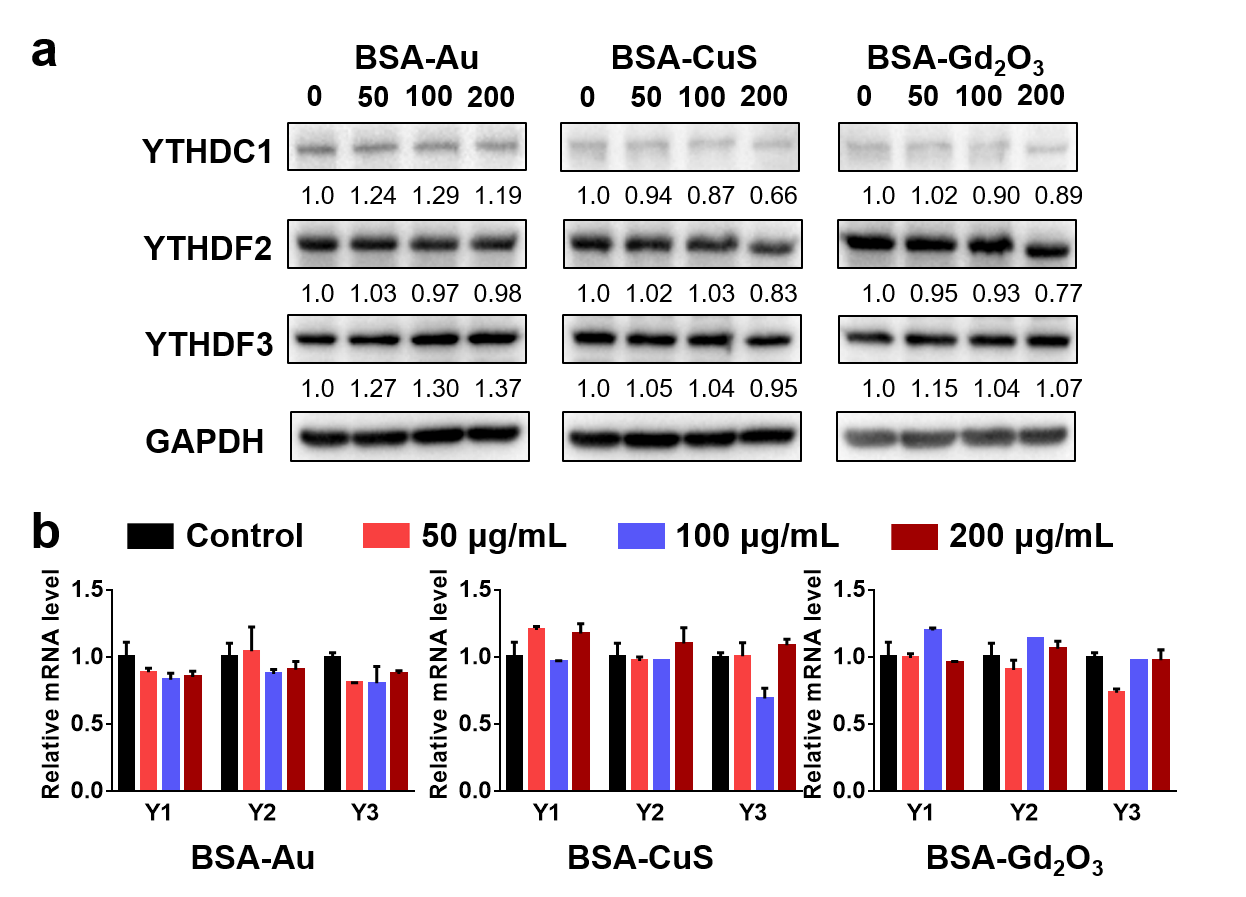


**Fig. S18** BSA-templated NPs induced changes of YTHDC1, YTHDF2 and YTHDF3. (a) Western blot analysis of YTHDC1, YTHDF2 and YTHDF3. A total of 30 μg protein was loaded (GAPDH was used as a control for loading). (b) Relative mRNA levels of YTHDC1, YTHDF2 and YTHDF3. Data are represented as the mean ± SD from three independent biological replicates. In all graphs, the control group was set to 1 and nanoparticles were expressed as the fold change relative to control.
